# Research on Quantitative Model of Brand Recognition Based on Sentiment Analysis of Big Data

**DOI:** 10.3389/fpsyg.2022.915443

**Published:** 2022-05-12

**Authors:** Lichun Zhou

**Affiliations:** School of Media and Communication, Shangqiu Normal University, Shangqiu, China

**Keywords:** brand recognition, quantitative model, sentiment analysis, big data, sentiment dictionary construction

## Abstract

This paper takes laptops as an example to carry out research on quantitative model of brand recognition based on sentiment analysis of big data. The basic idea is to use web crawler technology to obtain the most authentic and direct information of different laptop brands from first-line consumers from public spaces such as buyer reviews of major e-commerce platforms, including review time, text reviews, satisfaction ratings and relevant user information, etc., and then analyzes consumers’ sentimental tendencies and recognition status of the product brands. This study extracted a total of 437,815 user reviews of laptops from e-commerce platforms from January 1, 2019 to December 31, 2021, and performed data preprocessing on the obtained review data, followed by sentiment dictionary construction, attribute expansion, text quantification and algorithm evaluation. This paper analyzed the information receiving and processing hierarchy of the quantitative model of brand recognition, discussed the interactive relationship between brand recognition and consumer sentiment, discussed the brand recognition bias, style and demand in the context of big data, and performed the sentiment statistics and dimension analysis in the quantitative model of brand recognition. The study results show that the quantitative model of brand recognition based on sentiment analysis of big data can transform and map the keywords in text to word vectors in the high-dimensional semantic space by performing unsupervised machine learning on the text based on artificial neural network computer bionic metaphors; the model can accumulate each brand-related buyer review in the corresponding brand recognition dimension, so as to obtain the value of each product in each dimension of brand recognition; finally, the model will add the values of each dimension of brand recognition, that is, obtain the relevant value of the sum of each brand recognition. The results of this paper may provide a reference for further research on the quantitative model of brand recognition based on sentiment analysis of big data.

## Introduction

Brand recognition refers to the degree to which consumers are aware of a particular brand, which is the ability of consumers to identify a certain product category under different conditions, and can make consumers perceive or recall that a certain brand belongs to a certain type of product or service. The breadth and breadth of brand recognition can help identify the difference between a certain brand and competing brands (Van Grinsven and Das, 2016). Consumers’ recognition of commodities generally needs to be stimulated by the external environment to attract consumers’ attention. Consumers process, re-process and re-memorize commodity information with their personal knowledge and experience to form various interests of consumers on commodities (Amatulli et al., 2016). Sentimental focus includes the sensitivity and focus range of an individual to oneself, others and social life and sentimental response is the physiological and psychological reactions produced by sentiment in brand recognition activities. For product brands, recognition and affection are generally considered to be the most important brand associations (Lee, 2018). The former represents the beliefs and knowledge projected or formed by the stakeholders on the various attributes and characteristics of the commodity; the latter represents the attitude and feeling of the commodity projected by the stakeholders or based on the past experience related to the city. It can be said that recognition and sentiment are the two core means of measuring a brand, and the basis for generating the two and the objects of their effect are the various branch dimensions of the brand (Sousa et al., 2018).

In the era of big data, potential customers often use various forums, communities, evaluations, websites with good reputation and other platforms to learn about relevant product information, gain recognition of product brands, and use other users’ experience to judge whether the product meets its own requirements, and then decides whether to consume the product brand (Shayaa et al., 2018). However, brand recognition sentiment in the big data environment has rich characteristics, and the sentimental dimension must cover the sentimental level, attention level, sentimental level of the brand recognition subject, and the influence scope of brand recognition events (Kauffmann et al., 2020). The above and more elements should be considered when dividing the sentimental dimension; therefore, the sentimental dimension is divided into sentimental level, sentimental orientation, sentimental focus, sentimental arousal, sentimental transformation, sentimental response, sentimental warning (Behera et al., 2021). The influence of sentiment analysis can be continued throughout the creative process, ultimately affecting the overall operation of brand perception and the formulation of creative strategies. From the perspective of creative operation, sentiment analysis of big data splits and reorganizes elements according to the original materials provided by the demander, which reduces the overall dimension of creative content to the element level. In the first category, sentiment analysis is used to analyze the relationship between online reviews and product sales; the second category includes literature research on product evaluation based on sentiment analysis method (Moreno and Redondo, 2016).

This paper takes laptops as an example to carry out research on quantitative model of brand recognition based on sentiment analysis of big data. The basic idea is to use web crawler technology to obtain the most authentic and direct information of different laptop brands from first-line consumers from public spaces such as buyer reviews of major e-commerce platforms, including review time, text reviews, satisfaction ratings and relevant user information, etc., and then analyzes consumers’ sentimental tendencies and recognition status of the product brands. This study extracted a total of 437,815 user reviews of laptops from e-commerce platforms from January 1, 2019 to December 31, 2021, and performed data preprocessing on the obtained review data, followed by sentiment dictionary construction, attribute expansion, text quantification and algorithm evaluation. This paper analyzed the information receiving and processing hierarchy of the quantitative model of brand recognition, discussed the interactive relationship between brand recognition and consumer sentiment, discussed the brand recognition bias, style and demand in the context of big data, and performed the sentiment statistics and dimension analysis in the quantitative model of brand recognition. The detailed chapters are arranged as follows: Section 2 introduces data acquisition and research methods; Section 3 is results and their analyses; Section 4 is discussion; Section 5 is conclusion.

## Data Acquisition and Research Methods

### Data Collection

This paper takes laptops as an example to carry out research on quantitative model of brand recognition based on sentiment analysis of big data. The basic idea is to use web crawler technology to obtain the most authentic and direct information of different laptop brands from first-line consumers from public spaces such as buyer reviews of major e-commerce platforms, including review time, text reviews, satisfaction ratings and relevant user information, etc., and then analyzes consumers’ sentimental tendencies and recognition status of the product brands. This paper extracted 437,815 user reviews of laptops from the e-commerce platform from January 1, 2019 to December 31, 2021, and performed data preprocessing on the obtained review data. The data preprocessing process includes data de-duplication based on database technology, language segmentation and part-of-speech tagging, and stop word filtering and finally a total of 297,264 refined user review data were obtained. Since the amount of refined data is still relatively large and it is impractical to label all of them, 10% (29,726) of data was randomly select from the dataset and was divided into training data and test data and the remaining 267538 pieces of data are used as forecast data. Users’ experience and feedback on product quality on e-commerce platforms and other online transactions and social platforms are collected through data mining methods; consumers’ sentimental tendencies and recognition of laptop brands are obtained through repeated data cleaning, different quality evaluation clustering. This paper uses the gradient distribution model of brand recognition and the theory of brand recognition elements to make statistics and analysis from the four basic elements of recognition, respect, relevance and difference.

### Sentiment Dictionary Construction

Sentiment dictionary method is a commonly used text analysis method, which identifies the sentimental words in the text and calculates these words to determine the sentimental tendency of the text. Sentimental words are expressions of sentimental tendencies, polarities and evaluations of sentimental subjects and identifying subjective sentimental sentences according to sentimental words is determined by its own sentimental tendencies. The sentiment corpus contains all kinds of sentiments, including positive sentiment class, negative sentiment class and no sentiment class, in which no sentiment can express a neutral or rational state of the text, so this paper divides the non-sentiment category into neutral sentiment, forming three sentiment categories of positive sentiment, neutral sentiment and negative sentiment, corresponding to different sentiment classifications (Tritama and Tarigan, 2016). The word segmentation software is used to segment the experimental review text, and mark the effective word segmentation results by part of speech, such as words and phrases such as popular words on the Internet. The corpus not only classifies sentiment from sentiment words (adjectives, verbs, interjections), but also explicitly classifies inconspicuous network phrases to improve the efficiency of sentiment classification.

### Attribute Extension

The research method of intelligent semantic data mining based on word vector is the current cutting-edge intelligent semantic data mining technology. It transforms and maps the keywords in the text into word vectors in a high-dimensional semantic space by performing unsupervised machine learning based on artificial neural network computer bionic metaphors on the text. Based on this research method, the distinction and connection between the meanings referred to by keywords can be obtained by quantitatively measuring the geometric relationship between the corresponding word vectors. Compared with the traditional word frequency statistics method, the intelligent semantic data mining research method based on word vector can extract the core information in the text more deeply. On the basis of the previous successful application research, this paper will continue to use the word vector-based intelligent semantic data mining research method to mine the brand information of laptops in big data such as buyer reviews of e-commerce platforms.

### Text Quantization

First, unsupervised deep machine learning based on artificial neural network computer bio-mimetic metaphor is performed on all buyer reviews, thereby establishing a high-dimensional semantic space of word vectors. The weighted value is based on the projection of the topic contained in the buyer’s review on the description text of each dimension of brand recognition in the high-dimensional semantic space of the word vector, and the weighted value is based on the sentiment value of the buyer’s review. The weight is weighted and accumulated, so as to obtain the accumulation of the brand recognition of the notebook computer in each dimension of the buyer’s review.

Assuming that all the reviews of a product is put in the set *A* = {*a*_1_, *a*_2_, *a*_3_, …, *a*_*n*_}, and a set of sentiment words from the sentiment dictionary is *B* = {*b*_1_, *b*_2_, *b*_3_, …, *b*_*m*_}, then the online review data is an *N* × *M* matrix *C* = [*c*(*a*_*i*_, *b*_*j*_)]*_*ij*_*, where *c*(*a*_*i*_, *b*_*j*_) is the frequency of the sentimental word *b*_*j*_ in the online review *a*_*i*_ in the website. Assuming that the sentimental words in the online reviews are *D* = {*d*_1_, *d*_2_, *d*_3_, …, *d*_*k*_}, the probabilistic latent semantic analysis model calculates the sentimental words appearing in the online reviews to obtain the probability of the sentimental words of each online review. For the frequency *c*(*a*_*i*_, *b*_*j*_) of sentimental words in online reviews, it is defined as a conditional independent multinomial distribution:


(1) $$P\,\left(a,b\right)=\sqrt{a_{i}\cdot b_{i}}\cdot \sqrt{\frac{\sum_{i}^{n}\sum_{j}^{m}{\left(P_{a_{i}}-P_{b_{i}}\right)}^{2}}{\left(n-1\right)\,\left(m-1\right)}}$$

In this formula, the conditional probabilities *Pa*_*i*_ and *Pb_*i*_*, respectively, represent the probability of online review *a* and sentiment word *b* to generate latent sentiment word *d*, and online review *a*_*i*_ and sentiment word *b*_*j*_ are independent of the generation of latent sentiment word *d*.

Secondly, the corresponding brand recognition dimensions of each global Internet buyer’s reviews related to all laptops are accumulate on a yearly basis, so as to obtain the value of each laptop in each dimension of brand recognition in that year. Finally, the values of each notebook computer in each dimension of brand recognition are to be added in that year to obtain the relevant value of the sum of each brand recognition.

### Algorithm Evaluation

Random sampling selects a certain proportion of training corpus, performs manual back-to-back scoring, and compares the scoring result with the original satisfaction score to determine the proportion of noise. If the ratio is too high, it is necessary to increase the screening and elimination of noise corpus in the early stage; if the ratio is low; the noise corpus will not affect the machine learning effect in the later stage, and can be directly used for analysis in the next stage. Sentiment analysis uses neural networks to convert text evaluations that have been segmented into vectors, so that computers can read and learn. In the process of transformation, the column index is the eigenvector. Feature extraction refers to the selection of a representation method that can represent the target structure, and then has classification and judgment.

Suppose *e* is an information node describing a brand, and *f* is the best-selling brand in the industry, that is, *f* is a well-known brand of this type of product. Therefore, the calculation method of brand recognition can be converted into the calculation of the similarity *Q*(*e*, *f*) between *e* and *f*:


(2) $$Q\,\left(e,f\right)=\sqrt{e^{2}-f^{2}}-\frac{2\,R\,\left(e,f\right)}{\left|e\right|-\left|f\right|}$$

In the formula, *R*(*e*, *f*) is the string edit distance between *e* and *f*, and a threshold *g* is selected. When *R*(*e*, *f*) ≥ *g*, *e* is considered to be a well-known brand, otherwise, *e* is a general brand.

On the basis of converting the previous text into vectors, this paper uses the machine learning neural network algorithm to build the model. In terms of scoring standards, although the dichotomy method is simpler and the discrimination of machine learning is simpler, it also loses some information. Based on this, the research group uses the quintile method to enable machine learning to obtain more information, further refine the sentimental level of the text, and make the output results more interpretable.

## Results and Analysis

### Information Receiving Hierarchy of the Quantitative Model of Brand Recognition

**FIGURE 1:**
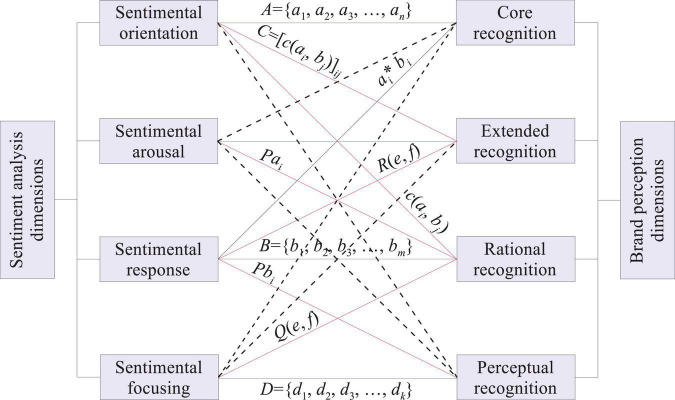
Text quantization and algorithm evaluation in the information receiving hierarchy of the quantitative model of brand recognition based on sentiment analysis of big data.

In the era of big data, consumers are more engaged in consumption activities as scattered subjects and they are accustomed to gaining recognition of a certain brand through online shopping, forums, communities, etc. The quantitative model should make full use of big data to analyze the usage habits of consumers, no longer regard consumers as passive individuals, improve consumers’ overall recognition of corporate brands in all aspects, and increase consumers’ stickiness to product brands. Secondly, consumers pay attention to the experience of virtual communities and they can participate in some virtual brand communities by browsing, posting, replying, etc., which can deepen their understanding of the brand, obtain the required information and resources, express their opinions and opinions, and can also use the community to communicate with other users who also like such brands (Davtyan et al., 2016). Moreover, users now also pay attention to the degree of fit between the values and outlook on life conveyed by the brand and their own concepts. The stronger the fit is, the stronger the stickiness of consumers to the company. In the era of big data, consumers’ recognition of and dependence on brands is gradually decreasing. Various forums, evaluations and other platforms can provide consumers with relevant product information, and they can judge whether the target product whether meet their standards, and then decide whether to buy, not just look at the brand without being based on the experience of other users. Figure 1 shows the text quantization and algorithm evaluation in the information receiving hierarchy of the quantitative model of brand recognition based on sentiment analysis of big data.

The quantity, quality, credibility and intensity of negative sentiments all have a significant negative impact on brand recognition, which is consistent with the conclusions of existing literature research. Among them, the number of reviews in negative sentiments is the primary consideration factor for consumers to make online purchases and read reviews, followed by the strength and quality of negative sentiments, while credibility has the least impact on brand perception. The sentimental resonance brought by this kind of understanding is positive and positive; after using the product and recognizing the value of the product, they will buy it again and become a loyal consumer. At present, researches on economy chain hotel brands mainly focus on brand evaluation and brand building. Because the number of negative sentiments is often affected by the conformity effect when it is transmitted to consumers, there is an interactive interference between brand recognition and conformity effect, especially the social identity in brand recognition is also affected by the conformity effect. For consumers with high brand recognition, the quality, credibility and strength of negative sentiments had a greater negative impact on brand perception; the opposite was true for consumers with low brand recognition. Therefore, brand recognition has a significant moderating effect on the relationship between negative sentiment and brand recognition, which also explains that e-commerce platforms have more negative sentiments, while consumers still have undiminished online shopping behaviors.

Consumers’ perceptions of brand rigid factors, especially functional factors, are often difficult to change once established. Therefore, once a brand occupies a certain functional factor, it can gain a certain monopoly advantage in the market and effectively prevent the entry of other brands, that is to say, the rigid factor has certain exclusive characteristics. With the continuous development of product diversification, hard factors can no longer cause absolute differences in brands, and soft factors based on sentimental interests begin to play an increasingly important role (Shirdastian et al., 2019). The degree of brand recognition can be divided into three levels, which can be specifically represented by the brand recognition pyramid. The first is brand recognition, which is the lowest level of brand recognition; the second is brand memory, which is a higher level than brand recognition and is based on consumers’ own memory. Big data enables brands to quickly and accurately measure the short-term response of audiences to marketing campaigns. However, there are dangers in making marketing decisions based solely on this information. Consumers often do not pay attention to the credit rating of the reviewer, nor do they trace the true reliability of the previous credit rating. As consumers get to know the brand products they come into contact with, consumers begin to pay attention to the brand, but there is still a jump from noticing that they have a clear understanding of the product’s features, functions, values, and features.

### Information Processing Hierarchy of the Quantitative Model of Brand Recognition

**FIGURE 2:**
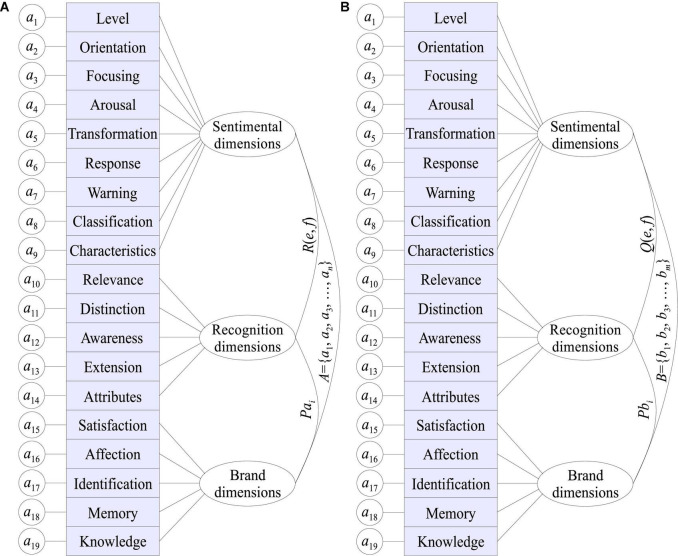
Information receiving and processing hierarchy of the quantitative model of brand recognition based on sentiment analysis of big data **(A)** Information receiving hierarchy, **(B)** Information processing hierarchy.

In the quantification model of brand recognition, the network recognition sub-layer refers to the ability to recognize the network itself, and can dynamically extend the recognition ability to the recognition of network resources, thereby improving the utilization of network resources and enhancing the dynamics of the network system. By using new productions, the memory burden on consumers is reduced, and decision-making is accelerated by eliminating the time needed to retrieve knowledge. Each time a new production is produced, it does not replace the original more detailed production, but both productions will be completed in the matching process. This recognition ability can help determine adjustment strategies and enhance the intelligent decision-making ability of survivable systems in various environments; at the same time, service ability recognition can dynamically discover and reasonably allocate goals and resources, and use memory, learning, reasoning, etc. As shown in Figure 2, the recognition unit structure can adjust its own behavior, topology and service parameters in time with the changes of the internal and external environment, task requirements and security threats of the survivable system. Just as brands promote consumer behavior by enhancing consumers’ satisfaction, self-esteem, and prestige, brands follow a similar path, with their associated attributes and benefits, promoting the public, residents, and decision-makers alike to generate a variety of perceptions of goods (Alaei et al., 2019).

Recognition theory explains the general laws that recognition subjects follow when they recognize objects. Recognition value is the overall evaluation of the various interests that the recognition subject has on the perceived recognition subject in the process of recognition, which is a measure of consumers’ recognition of brand value. Brand recognition value should include two aspects, one is the overall evaluation of the various benefits consumers perceive the brand, and the other is the consumer’s recognition value. Consumer perception and evaluation of brand benefits are the main content of brand recognition value. From the perspective of the composition of brand value, consumers’ recognition of brands can be divided into core recognition and extended recognition; consumers’ recognition can be divided into rational recognition and perceptual recognition. Therefore, core recognition and extended recognition constitute one dimension of the brand matrix, and rational recognition and perceptual recognition constitute another dimension of the brand matrix. The intersection of these two dimensions can divide the components of the brand into four spaces and each space is a collection of elements that make up various aspects of the brand. When operating the brand recognition value, it is to observe whether each quadrant of the matrix has rich content in combination with the brand matrix. The corresponding brand recognition element in which quadrant is lacking in content is the object of corporate strengthening.

When faced with a problem to solve, such as brand choice, individuals will look for available productions that match current goals and conditions. If such a production is found, it will be executed to solve the problem and the situation becomes complicated when such productions do not exist, such as dealing with a new domain. Customers learn declarative knowledge when incoming external knowledge is encoded and added to the associative network of facts. In general, when a consumer starts to use a product, customers will need to learn a lot of product-related knowledge, after purchase after purchase, through the interpretive application of the above-mentioned declarative knowledge, production editing, and production adjustment, and they are able to expend decreasing recognition effort when choosing a brand (Martí-Parreño et al., 2017). The sub-layer of service recognition refers to the recognition of the system task environment and service capability, involving the perception of complex internal and external environment, the recognition of the rationality of the matching between the environment and the task, and the consistent recognition among multiple tasks. The connections between new knowledge units in the brain and existing ones, and the organization of each knowledge unit in the knowledge network determine the level of knowledge extraction and information efficiency.

## Discussion

### Interactive Relationship Between Brand Recognition and Consumer Sentiments

**FIGURE 3:**
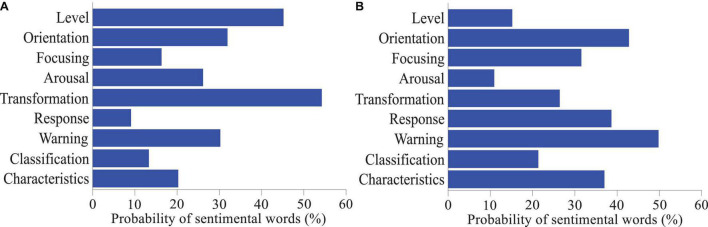
Probabilities of sentimental words in information receiving **(A)** and processing **(B)** hierarchy of the quantitative model of brand recognition based on sentiment analysis of big data.

The sentiment dimension is based on sentiment theory and there is an internal mechanism correlation between various sentiment dimensions in the network environment. The sentimental level can be progressive or jumping from one layer to another and the sentimental orientation generally reflects the changing trend of social brand recognition events related to one knowing the development trend. When consumers learn product information, they have to pay a certain cost in the process of perception, processing and reprocessing of commodity information, including monetary cost, time cost, energy cost and other substitution costs. Different sentimental focus ranges have different sentimental responses, reflecting different hidden sentiments and the degree of sentiment can appropriately turn recessive sentiment into explicit sentiment (Sheela, 2016). The influence of sentimental arousal brand recognition environment on sentimental individuals is generally from the activation of irrelevant sentiments or low-level sentiments to the investment of high-level sentiments (Figure 3). Sentimental conversion refers to the conversion between different sentiments under certain conditions and at a certain speed under brand recognition. Sentimental early warning is a comprehensive qualitative target dimension based on sentimental dimensions such as sentimental level, sentimental orientation, sentimental response, and sentimental focus. In addition, the influencing factors and the degree of relationship in the sentimental dimension are interconnected, and changes in some dimension elements will cause changes in other elements.

As a symbolic attribute of the brand, brand personality is also the object of consumers’ recognition of the brand; these three dimensions can also be introduced into the brand personality for consideration. The recognition strength of brand personality refers to the ability of consumers to personify the brand and then recognize a brand personality, which is directly affected by the marketing stimulus of the enterprise. Brand personality uniqueness refers to the extent to which consumers perceive a brand’s personality as distinct and distinctive (Yousaf et al., 2021). Brand personality identity refers to the degree to which consumers believe that their desired personality matches the brand’s personality. For each recognition object attribute, consumers will evaluate it from three aspects: the recognition strength of the attribute, the reputation of the attribute and the uniqueness of the attribute. Among them, the intensity of recognition refers to the extent to which consumers perceive the existence of this attribute, which is a recognition variable. Brand reputation is a sentimental variable, which describes the degree to which an attribute is distinct and distinctive. Therefore, this is a recognition variable with some sentimental factors, but it is still a recognition variable in general.

In the process of processing brand information, consumers will undergo a series of psychological and behavioral changes. In the mass consumer goods market, the products or services provided by competitors are not very different in function and quality. At this time, consumers often make purchasing decisions based on their familiarity with the brand and the amount of association information and brands help consumers establish relevant associations with the help of product quality, marketing behavior, and information transmission. Brand associations enhance consumers’ familiarity with brands, seek to match consumers’ values, and ultimately become consumers’ understanding, memory, association and identification. Therefore, in the product selection stage, brand association is an indispensable factor in the process of consumers forming brand recognition. When consumers agree with the brand’s development concept, their perception of product function and durability will be improved, and they will have a certain tolerance for the company’s product premium behavior. Some scholars have pointed out that consumers evaluate products based on their perception of price, quality and value, rather than the objective attributes of products (Kang and Moon, 2017). Therefore, brand recognition can effectively improve consumers’ perception of product quality. In the post-purchase stage, consumers build sentimental resonance by understanding the brand, recognizing the brand value, and ultimately promoting repeat purchase behavior.

### Brand Recognition Bias, Style and Demand in the Context of Big Data

Accurate positioning and flexible adaptation are crucial to the development of brand recognition quantification, but they are far from enough. If a brand wants to succeed, it must have a long-term strategic plan. The research on sentiment analysis and products based on big data can be divided into two categories: product sales factor analysis based on sentiment analysis method and product evaluation based on sentiment analysis method. The sentimental dimension of traditional psychology mainly includes the dimensions of sentiment type, sentiment conversion and sentiment arousal. The creative elements are summarized by category, combined with arrangement and combination, manual screening, and real-time optimization in use, so that the creative plan can meet the staged and diversified demands of brand advertising. Table 1 shows the statistics of sentimental classification of the laptop brand based on big data. In this sentiment analysis process, the relevant data of each delivery will be retained and become a key indicator to guide the follow-up content, helping to build a long-term brand development strategy. Text sentiment analysis, also known as opinion recognition, opinion mining, etc., refers to the analysis process of identifying, extracting, classifying, summarizing and reasoning about opinions, sentiment polarity, subjectivity and objectivity in texts, among which sentiment classification is the most common application, whose main task is to perform sentiment classification on subjective texts (Rehman et al., 2019).

**TABLE 1:** Statistics of sentimental classification of the laptop brand based on big data.

Online review *a_*i*_*	Sentimental word *b_*j*_*	Positive sentiment		Neutral sentiment		Negative sentiment	
		
		Probability	Proportion	Probability	Proportion	Probability	Proportion
0.05	1.00	23.53%	37.34%	82.27%	21.55%	20.87%	66.41%
0.10	0.95	75.54%	57.35%	26.46%	31.15%	14.04%	30.57%
0.15	0.90	13.34%	84.64%	86.13%	64.73%	36.14%	27.64%
0.20	0.85	4622%	46.17%	54.03%	64.54%	22.53%	32.45%
0.25	0.80	63.76%	25.07%	33.44%	35.32%	69.16%	46.53%
0.30	0.75	15.85%	16.65%	56.03%	24.46%	57.09%	46.57%
0.35	0.70	83.07%	11.44%	37.75%	75.64%	35.05%	17.44%
0.40	0.65	10.45%	45.26%	25.86%	64.47%	57.03%	64.88%
0.45	0.60	46.81%	74.80%	74.75%	35.35%	63.02%	85.63%
0.50	0.55	44.15%	35.43%	44.75%	55.07%	24.63%	40.43%
0.55	0.50	27.23%	97.49%	25.03%	34.73%	24.89%	35.34%
0.60	0.45	56.93%	36.38%	36.99%	25.38%	68.81%	27.53%
0.65	0.40	18.67%	66.11%	53.14%	75.69%	64.34%	76.96%
0.70	0.35	85.35%	35.27%	86.06%	69.21%	36.84%	65.44%
0.75	0.30	45.18%	21.63%	77.36%	43.76%	24.81%	55.34%
0.80	0.25	33.04%	77.53%	95.06%	66.35%	44.78%	48.70%
0.85	0.20	29.54%	53.02%	34.04%	48.11%	57.35%	36.44%
0.90	0.15	67.23%	25.75%	43.73%	71.32%	34.67%	74.83%
0.95	0.10	54.53%	24.34%	72.04%	24.64%	89.66%	36.03%
1.00	0.05	75.81%	63.19%	46.75%	30.58%	37.74%	76.43%

**FIGURE 4:**
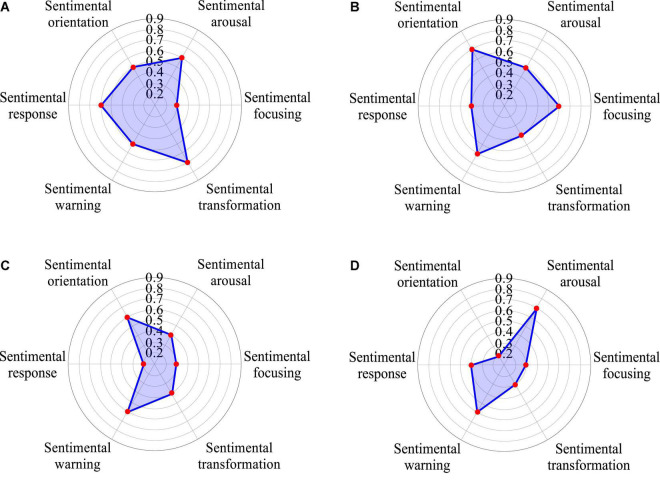
Brand recognition bias, style and demand in quantitative model of brand recognition based on sentiment analysis of big data **(A)** Features, **(B)** Appearance, **(C)** Experience, **(D)** After-sales service.

As shown in Figure 4, building a successful brand includes four stages of brand recognition, brand recognition, brand association, and brand loyalty. The brand should have a relatively high reputation, and then the audience should have a relatively full understanding of the brand’s connotation and personality. The sentimental resonance brought by this kind of understanding is positive and positive; after using the product and recognizing the value of the product, they will buy it again and become a loyal consumer. At present, researches on economy chain hotel brands mainly focus on brand evaluation and brand building. Therefore, brand recognition is an important link in the construction process, and it plays a key role in cultivating loyal consumers and improving brand benefits, especially when many brands appear in the same product (Chan and Chong, 2017). The amount of negative sentiment creates sensory stereotypes in consumers in the first place and the credit rating of the reviewer is based on the reviewer’s previous consumption records to grade their credit, etc. It is the highest level of brand recognition and the most important indicator to measure the psychological and sentimental share of a brand and recognition is the consumer’s recognition of the brand, which is in the fourth stage of the brand. Actions such as price promotions, which are detrimental to long-term brand equity, confuse this measurement, so a program built on measurement results may end up downgrading the brand.

In terms of quality perception, extroverted and open consumers seem to be more willing to accept and understand things because they have a positive bias in quality perception; the most closed are sensitive consumers, who are the most conservative in a group of consumers whose quality recognition is lower than the average consumer recognition; agreeable and cautious consumers are in the middle, but their recognition of performance is higher, although not significantly. In terms of brand associations, extroverted consumers are more sensitive to innocence and often ignore other aspects of personality; agreeable consumers are indeed more agreeable because they feel most brands are competent; cautious consumers are harmonious when the nurturing type of brand personality is linked; the sensitive type of consumer is too sensitive when they think these brands are rough; the open type consumer is perhaps the most forgiving and their perception is closest to the mean, thinking that everyone is similar (Kilei et al., 2016). After controlling the brand personality variables, the correlation between consumer personality and quality recognition can be clearly seen. The correlation between extroversion factor and applicability recognition is very significant, which shows that extroverted consumers pay more attention to the applicability of products.

### Sentiment Statistics and Dimension Analysis in the Quantitative Model of Brand Recognition

**FIGURE 5:**
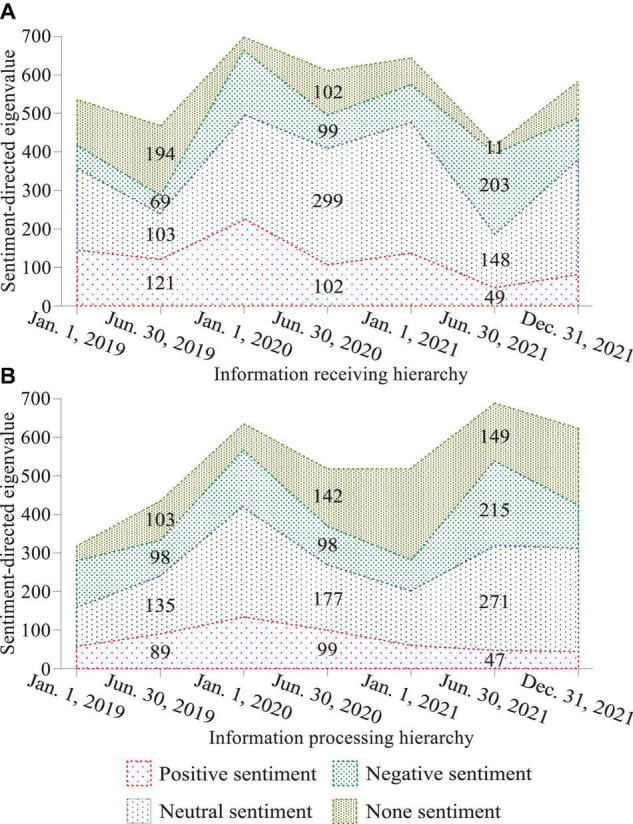
Sentiment statistics and dimension analysis of information receiving **(A)** and processing **(B)** in the quantitative model of laptop brand recognition based on sentiment analysis of big data.

In the era of mobile internet, after consumers have brand recognition of a specific product, consumers with different sentimental tendencies will release evaluation information through many communication platforms, resulting in the rapid generation of sentimental recognition of the brand, which is huge in number and in various forms. The result contains a lot of user information, which makes the network evaluation environment take on the form of big data, hereinafter referred to as evaluation big data (Figure 5). In the online evaluation big data environment, it is difficult to directly quantify consumer sentiment; quantifiable evaluation information interacts and influences each other, and jointly promotes the dissemination of online evaluation. First, consumers with different sentimental inclinations publish different evaluation information, but their sentiments can be inferred from the evaluation information they publish; secondly, consumers browsing the evaluation information will affect their sentimental tendencies, and then the sentimental evolution is affected by the evaluation information; third, a large number of consumers with different sentimental inclinations publish their own opinions and participate in discussions, which will lead to the interaction of brand evaluation information. In the evaluation big data environment, the state and degree of consumer sentimental evolution can be inferred and perceived by studying the interaction degree of online evaluation information of brand recognition. Therefore, in the big data environment, the evolution trend of consumers’ sentiments can be studied through the interaction of brand evaluation information, and the degree of mutual influence between various sentiments can be inferred (Seo, 2016).

Faced with many brands in life, it is impossible for every consumer to pay attention to all brands, only those brands that are related to the current needs, interests, and expectations of target consumers, as well as new, unique, strong, and other advertising contrasting brand messages to be noticed. Consumers’ processing of brand information presents two routes starting from their attention, which is the shallow processing route, that is, the brand information arouses the unintentional attention of consumers. Due to the low degree of involvement of consumers, it is impossible to process the information in depth and detail, so only a shallow impression can be left in the mind to reach the level of recognition of brand recognition and the other is the deep processing route, that is, due to the outside world (Pejić Bach et al., 2019). The brand information is just related to the requirements and expectations of consumers, which arouses intentional attention, activates the existing knowledge and experience of consumers, and hopes to seek further explanations for the brand information, so as to leave a deep impression in the mind and achieve brand recall. From a psychological point of view, brand association is based on brand recognition. If the brand information that is unintentionally noticed is related to the current needs of consumers, this unintentional attention may be transformed into intentional attention, which will lead to deep processing by consumers, and finally reach the level of brand recall.

The brand builder constructs the corresponding brand recognition through the various dimensions of the brand, including recognition identity and affective identity, etc., and then relies on the relevant media to project the brand recognition into the subjective perception of the dissemination audience to generate the corresponding brand image, including recognition image and finally form the corresponding brand. Familiar information is easier to retrieve from memory and is considered more practical or relevant than unfamiliar information. Simply repeating a piece of information in the media regardless of its accuracy makes that information more accessible and thus falsely perceived as more accurate. Another bias in judgment comes from the availability of information, that is, consumers’ judgment of probability distribution depends on the difficulty of obtaining information (Bilgin, 2018). In the process of judgment, consumers usually give high weight to some information that is easy to obtain and remember. The consumers estimate the frequency or probability of uncertain events using the instances or relationships they can most easily think of. Since decision makers do not have complete rational ability, every time they make a decision, they will find out from memory the cases that are most similar to the current situation and the consequences of the corresponding decision, and then make judgments and decisions.

## Conclusion

This paper takes laptops as an example to carry out research on quantitative model of brand recognition based on sentiment analysis of big data. The basic idea is to use web crawler technology to obtain the most authentic and direct information of different laptop brands from first-line consumers from public spaces such as buyer reviews of major e-commerce platforms, including review time, text reviews, satisfaction ratings and relevant user information, etc., and then analyzes consumers’ sentimental tendencies and recognition status of the product brands. The brand builder constructs the corresponding brand recognition through the various dimensions of the brand, including recognition identity and affective identity, etc., and then relies on the relevant media to project the brand recognition into the subjective perception of the dissemination audience to generate the corresponding brand image, including recognition image and finally form the corresponding brand. A reviewer’s credit rating is based on the reviewer’s previous consumption records to grade their credit, etc. and consumers often do not pay attention to the reviewer’s creditworthiness, nor do they track the true reliability of previous creditworthiness. In the online evaluation big data environment, it is difficult to directly quantify consumer sentiment; quantifiable evaluation information interacts and influences each other, and jointly promotes the dissemination of online evaluation. The study results show that the quantitative model of brand recognition based on sentiment analysis of big data can transform and map the keywords in text to word vectors in the high-dimensional semantic space by performing unsupervised machine learning on the text based on artificial neural network computer bionic metaphors; the model can accumulate each brand-related buyer review in the corresponding brand recognition dimension, so as to obtain the value of each product in each dimension of brand recognition; finally, the model will add the values of each dimension of brand recognition, that is, obtain the relevant value of the sum of each brand recognition. The results of this paper may provide a reference for further research on the quantitative model of brand recognition based on sentiment analysis of big data.

## Data Availability Statement

The original contributions presented in the study are included in the article/supplementary material, further inquiries can be directed to the corresponding author/s.

## Author Contributions

The author confirms being the sole contributor of this work and has approved it for publication.

## Conflict of Interest

The author declares that the research was conducted in the absence of any commercial or financial relationships that could be construed as a potential conflict of interest.

## Publisher’s Note

All claims expressed in this article are solely those of the authors and do not necessarily represent those of their affiliated organizations, or those of the publisher, the editors and the reviewers. Any product that may be evaluated in this article, or claim that may be made by its manufacturer, is not guaranteed or endorsed by the publisher.
